# OCRClassifier: integrating statistical control chart into machine learning framework for better detecting open chromatin regions

**DOI:** 10.3389/fgene.2024.1400228

**Published:** 2024-12-04

**Authors:** Xin Lai, Min Liu, Yuqian Liu, Xiaoyan Zhu, Jiayin Wang

**Affiliations:** ^1^ School of Computer Science and Technology, Xi’an Jiaotong University, Xi’an, China; ^2^ Shaanxi Engineering Research Center of Medical and Health Big Data, Xi’an Jiaotong University, Xi’an, China

**Keywords:** cell-free DNA, open chromatin region, sequencing data analysis, machine learning approach, multivariate control chart, noisy label

## Abstract

Open chromatin regions (OCRs) play a crucial role in transcriptional regulation and gene expression. In recent years, there has been a growing interest in using plasma cell-free DNA (cfDNA) sequencing data to detect OCRs. By analyzing the characteristics of cfDNA fragments and their sequencing coverage, researchers can differentiate OCRs from non-OCRs. However, the presence of noise and variability in cfDNA-seq data poses challenges for the training data used in the noise-tolerance learning-based OCR estimation approach, as it contains numerous noisy labels that may impact the accuracy of the results. For current methods of detecting OCRs, they rely on statistical features derived from typical open and closed chromatin regions to determine whether a region is OCR or non-OCR. However, there are some atypical regions that exhibit statistical features that fall between the two categories, making it difficult to classify them definitively as either open or closed chromatin regions (CCRs). These regions should be considered as partially open chromatin regions (pOCRs). In this paper, we present OCRClassifier, a novel framework that combines control charts and machine learning to address the impact of high-proportion noisy labels in the training set and classify the chromatin open states into three classes accurately. Our method comprises two control charts. We first design a robust Hotelling T^2^ control chart and create new run rules to accurately identify reliable OCRs and CCRs within the initial training set. Then, we exclusively utilize the pure training set consisting of OCRs and CCRs to create and train a sensitized T^2^ control chart. This sensitized T^2^ control chart is specifically designed to accurately differentiate between the three categories of chromatin states: open, partially open, and closed. Experimental results demonstrate that under this framework, the model exhibits not only excellent performance in terms of three-class classification, but also higher accuracy and sensitivity in binary classification compared to the state-of-the-art models currently available.

## 1 Introduction

Open chromatin regions (OCRs) that are accessible to DNA regulatory elements, play a crucial role in cellular activities and gene expression (Thurman et al., 2012; Yao et al., 2015; Klemm et al., 2019). It is reported that OCR patterns are associated with many complex traits and diseases. For example, several cancers are observed unique patterns of open chromatin regions, which are considered valuable in underlying cancer development through epigenetic mechanisms. It is also suggested as potential biomarkers in cancer early-diagnosis (Ivanov et al., 2015; Flavahan et al., 2017). Thus, detecting OCRs and identifying their patterns is a basic computational problem in epigenetic research and their clinical applications.

The traditional methods for identifying OCRs (ChIP-Seq (Johnson et al., 2007), DNase-seq (Crawford et al., 2006), MNase-seq (Schones et al., 2008), FAIRE-seq (Giresi et al., 2007), ATAC-seq (Buenrostro et al., 2015)) are all complex experimental processes. Until recent decade, detecting OCRs from plasma cell-free DNA sequencing data becomes popular. Plasma cell-free DNA (cfDNA) molecules are short fragments generated through a non-random procedure (Ivanov et al., 2015; Gai and Sun, 2019; Van der Pol and Mouliere et al., 2019; Lo et al., 2021; An et al., 2023). Recent studies conducted by Snyder (Snyder et al., 2016) and Ulz (Ulz et al., 2016) have shown a correlation between the characteristics of cfDNA fragments and gene expression levels, which provide new insights on detecting OCRs. Snyder first proposed the windowed protection score (WPS) to established a whole-genome nucleosome landscape, which was able to transform the cfDNA sequencing data into waveforms whose peaks corresponded to nucleosomes (Snyder et al., 2016). Ulz presented EP (Expression Prediction) algorithm (Ulz et al., 2016), which focused on inferring gene expression levels by low sequencing coverage in OCRs. Sun found that the characteristics of cfDNA fragment distribution can be reflected by the differentially phased cfDNA fragment end signals (Sun et al., 2019). Based on these pioneering works, Wang proposed OCRDetector, which among the first approach introduces a machine learning framework (Wang et al., 2021). However, it could hardly overcome the limitations of artificially constructed features and it didn’t consider the noisy labels. Subsequently, Ren introduced OCRFinder (Ren et al., 2023), which first leverages deep learning for feature extraction and incorporates ensemble techniques and semi-supervised strategies to tackle noisy label learning. This approach has performed impressive results in various evaluations but the performance of the model also be affected by the high proportion of noisy labels in the training sets. Jin proposed utilizing OCF values and SVM classification model for predicting tissue injury (Jin et al., 2023), but this study relies on biological experiments to obtain known tissue-specific open chromatin regions, hence it is not possible to directly analyze the genome-wide chromatin accessibility using cfDNA-seq data. Clearly, current methods are limited to distinguishing between chromatin open and closed regions.

However, cfDNA data represents a mixed sample from multiple tissues, the chromatin states cannot be simplified as solely fully open or fully closed. Actually, in real sequencing data, we often observe the regions that seem partially open. We may call them partially open chromatin regions (pOCRs). For those pOCRs, none of the existing approach, whatever the machine learning-based ones or deep learning-based ones can achieve accurate detection. There are several reasons for this: the training data for the existing learning models only consists of two labels, where the genomic regions with active gene expression are categorized as open chromatin regions, while the regions with silent gene expression are categorized as non-open chromatin regions. It is a computational challenge to correctly mark the partially open chromatin regions, because those pOCRs are randomly assigned as either OCRs or non-OCRs. In addition, the threshold differentiate OCRs and pOCRs varies across samples, which may be influenced by sequencing coverage, local distribution of read depths, etc. Thus, a self-adaptive method is needed to identify three-class regions using training datasets that only contain two-class labels.

The patterns of cfDNA fragments is a random variable that varies along the genomic locus. If we map the reference genome to a X-axis, map the WPS scores to a Y-axis, then the patterns become a function on pseudo-time series. Statistical process control (SPC) is a family of methods that capture the statistical characteristics on time-series data which is a potential way of identifying the thresholds and differentiating the pOCRs from OCRs and non-OCRs. The three states of chromatin correspond to three distinct data distributions of cfDNA fragment features. Thus, let non-OCRs define as the normal state in the statistical process, then both the OCRs and pOCRs can be defined as abnormal states with different abnormal patterns, aligning precisely with the in-control and out-of-control states of a control chart. The computational problem switches to design a control chart that is able to efficiently report the out-of-control states. CUSUM is a control chart that has been widely used in a range of healthcare settings, from describing the learning curves of surgical or procedural skills (Waller and Connor, 2009; Je et al., 2015; Kim et al., 2015), to clinical audits (Cockings et al., 2006; Calsina et al., 2011), and quality-assurance studies (Novick et al., 2006; Hasegawa et al., 2017). Hotelling’s T^2^ is another control chart that has been widely used for measuring and monitoring biological and biomedical investigations (Lv et al., 2014; Ercan et al., 2015). To better use the control charts to detect open chromatin regions, we need to re-design it a little bit. For example, the T^2^ chart, assume that the in-control (IC) group is the sole population used for establishing a decision boundary. Nevertheless, this assumption has limited the advancement of more efficient control chart techniques that can leverage the available out-of-control (OC) information. A sensitized T^2^ chart (Park and Kim, 2019) is proposed later, wherein OC observations extracted from historical records were classified as “predefined OC,” while those arising from novel fault types not encountered previously were identified as “undefined OC.” Based on this, let OCR being the predefined OC and pOCR as undefined OC.

However, the classification of control charts is still subject to certain errors based on control limits. Therefore, to improve the recognition of multiple states of chromatin openness, this study then uses a CNN-based model under the framework of confident learning (Curtis et al., 2021). Nowadays, an increasing number of CNN-based models are being used in various fields. If an efficient encoding method is available, deep learning models have the capability to automatically extract the distinctive fragmentation patterns of cfDNA molecules at OCRs without requiring manual intervention. Consequently, employing a CNN-based model for OCR estimation is a viable and practical approach.

In this article, we proposed a novel computational approach, OCRClassfier, to accurately detect open chromatin regions together with the partially open chromatin regions from cfDNA sequencing data. A novel sensitized T^2^ control chart is designed to identify the candidate OCRs. This control chart integrates a MEWMA control chart with the OCRFinder noise-tolerance model. The control chart we designed considers two types of probabilities the first of which is derived from a MEWMA control chart, enabling the detection of mean shifts in any direction within the process. This aspect ensures robust monitoring capabilities. The second probability is based on a noise-tolerance machine learning method OCRFinder, effectively distinguishing between OC and IC data. Combining these probabilities enhances the accuracy and reliability of the control chart in accurately identifying the deviations in the process. This new control chart aims to effectively monitor chromatin states. By training the sensitized T^2^ control chart using data from OCRs and non-OCRs, we obtained a control chart capable of identifying OCRs, non-OCRs, and pOCRs. Thus, we transformed the original training dataset with only two labels into three categories. In order to obtain an improved control chart, we have also devised a robust Hotelling T^2^ control chart specifically designed to identify OCRs and non-OCRs. This helps in filtering out noisy labels from the training set and then can reduce the impact caused by such noisy labels. Additionally, it is worth mentioning that the obtained three-class training set still contains a small amount of noisy labels. Therefore, we conducted further training by using a neural network model under the confident learning strategy to minimize the impact of noisy labels.

## 2 Materials and methods

In the WPS waveform, peaks represent nucleosome positions, whereas peaks disappear in OCRs. In terms of sequencing coverage, there was a significant decrease in coverage of OCRs. In the end signals, the head and tail peaks represent both sides of the nucleosomes, and the end signals are also lost in OCRs.

**FIGURE 1:**
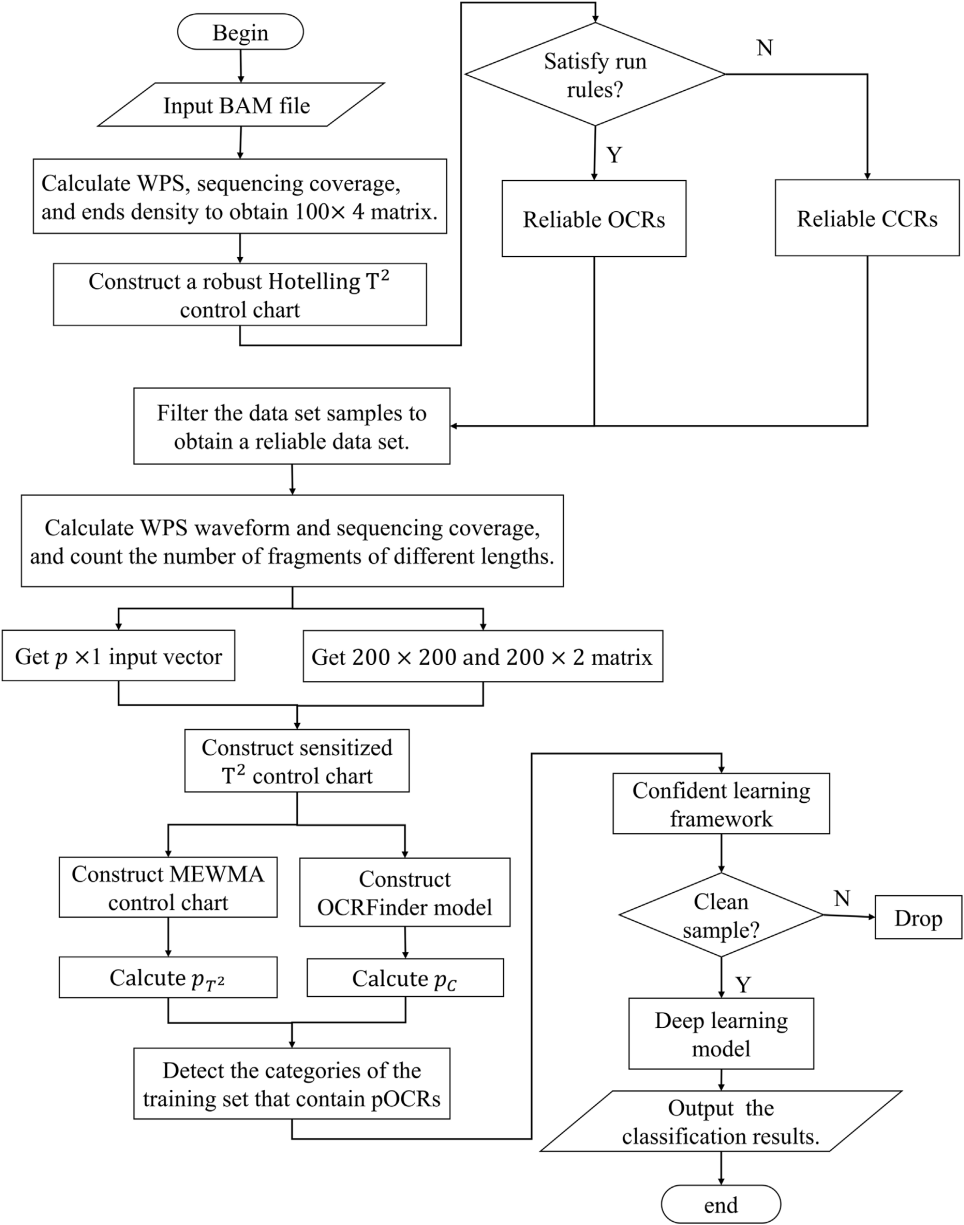
The flow chart of OCRclassifier.

Here, we present a three-stage multiclass classification algorithm that introduces the idea of multivariate control charts and machine learning. The three stages are data pre-processing, control charts designing and model co-teaching. The second step consists of two control charts: robust Hotelling T^2^ control chart and sensitized T^2^ control chart. The data pre-processing converts cfDNA-seq data into vectors and matrixes suitable for control charts and two-dimensional images suitable for neural networks, respectively. The second stage first uses robust Hotelling T^2^ control chart with minimum regularized covariance determinant (MRCD) to reduce the scale of noisy labels on the training set and then designs sensitized T^2^ control chart that combines multivariate exponentially weighted moving average (MEWMA) and OCRFinder classification algorithm to give the model an initial recognition ability. In the last stage, we use confident learning strategy to avoid the impact of noisy labels and classify the three open states of chromatin in whole genome regions. The framework of OCRClassifier model is shown in Figure 1.

### 2.1 Data processing pipeline

The cfDNA-seq data in fastq format is processed by BWA and Samtools. The input data of the control charts is not the same as the input data of the deep learning model.

The human genome is divided into multiple intervals (Wang et al., 2021), each containing 20 kilobase pairs (kbp). Subsequently, for each of these intervals, the WPS waveform, sequencing coverage, Uend signal, and Dend signal are computed. As mentioned above, the size of a single nucleosome is about 167 base pairs (bp), while the linked DNA connecting the nucleosome is about 20 bp in size (Struhl and Segal, 2013). Features showed strong periodic patterns, with one nucleosome per period of approximately 190 bp in length (Snyder et al., 2016). Thus, a sliding window of 200 bp size was defined in each 20 kbp interval, and the averages of the four features within the window were calculated to obtain a 100 × 4 input matrix as the input data of Hotelling T^2^ control chart.

**FIGURE 2:**
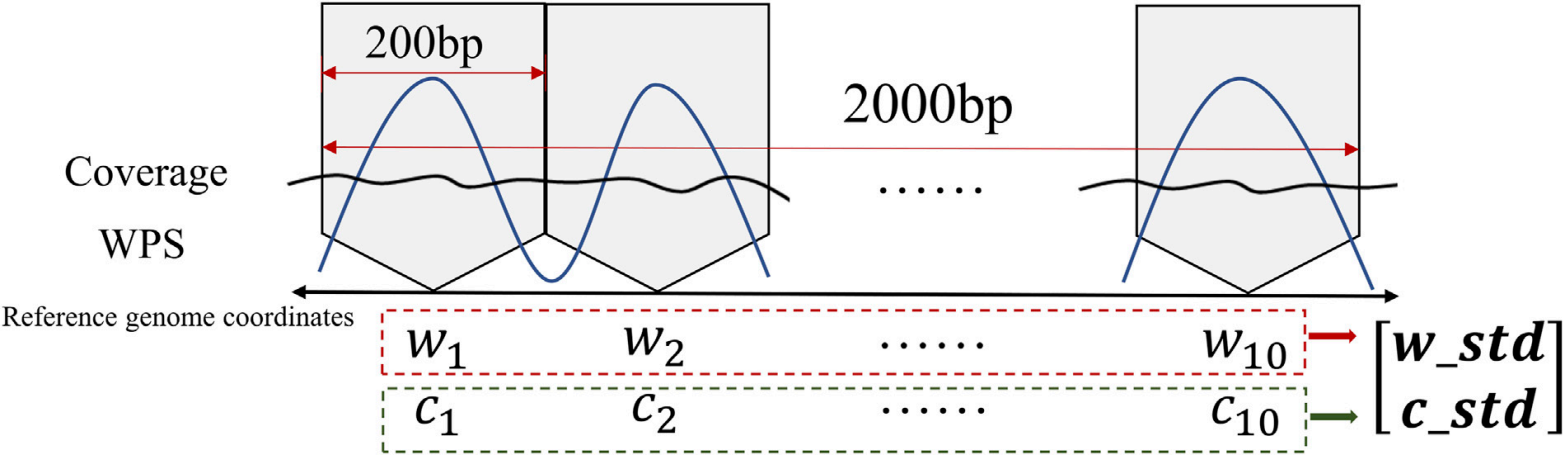
Illustration of input data processing for MEWMA control charts.

In order to apply the sensitized T^2^ control chart, the standard deviation of the windowed average values of the WPS and sequencing coverage features is calculated for each sample, the calculation process is shown in Figure 2. By doing so, the cfDNA-reads data is transformed into a vector representation. This enables us to assess the variability within the respective windows and gain insights into the fluctuation characteristics and dispersion patterns exhibited by these two specific attributes.

For deep learning model, the input data type is the same as OCRFinder. We convert cfDNA-reads data into two-dimensional matrixes T , the rows correspond to genomic coordinates, while the columns represent the lengths of cfDNA-reads. Moreover, sequencing coverage, WPS score, and the density of the head and tail of cfDNA fragments are encoded in a similar manner. This encoding process results in the generation of two-dimensional matrices, which serve as additional inputs to deep learning model.

### 2.2 Control charts designing

#### 2.2.1 Design robust Hotelling T^2^ control chart

Let $X_{1},X_{2},\ldots ,X_{n}$ be a dataset in which $X_{i}={\left[X_{i1},X_{i2},\ldots ,X_{ip}\right]}^{\prime }$ denotes the i -th observation ( i=1,2,…,n ; where n is the total number of samples to be monitored), p is the number of variables. Traditional Hotelling T^2^ control charts are monitored according to the Hotelling T^2^ statistic, which is calculated using Formula 1 (Hotelling, 1947):

$$T^{2}\left(i\right)={\left(X_{i}-\bar{X}\right)}^{\prime }S^{-1}\left(X_{i}-\bar{X}\right)$$ (1)

Where $\bar{X}$ and S are the mean and covariance matrix of the distribution, respectively. It can be seen from the formula that the key to calculating the statistics lies in the mean vector $\bar{X}$ and the covariance matrix S . However, the mean vector and covariance matrix are likely to be affected by outliers in the samples, which make the traditional Hotelling T^2^ control chart not robust and then obtain biased results.

In the problem of detecting OCRs in training sets, the number of outliers in the observed values may be large, which may have a great impact on the mean vector and covariance matrix, resulting in biased results. Thus, we use the MRCD estimation to construct a robust Hotelling T^2^ statistic (Formula 2).

$$T_{MRCD}^{2}\left(i\right)={\left(X_{i}-{\bar{X}}_{MRCD}\right)}^{\prime }S_{MRCD}^{-1}\left(X_{i}-{\bar{X}}_{MRCD}\right)$$ (2)

Here, ${\bar{X}}_{MRCD}$ and $S_{MRCD}$ are the robust mean vector and the robust covariance matrix obtained according to the MRCD estimation method (Boudt et al., 2020).

We assume that the distribution of $T_{MRCD}^{2}$ statistics is F distribution, then the mean and variance of $T_{MRCD}^{2}$ are defined as Formula 3 and 4

$$E\left(T_{MRCD}^{2}\right)=d\frac{q}{q-2}$$ (3)

$$Var\left(T_{MRCD}^{2}\right)=d^{2}\frac{2q^{2}\left(p+q-2\right)}{p\left(q-4\right){\left(q-2\right)}^{2}}$$ (4)

Thus d and q are calculated, from which the control limit UCL is found, shown in Formula 5. $\hat{d}$ , $\hat{q\;}$ is the numerical solution of the mean and variance of $T_{MRCD}^{2}$ calculated by Monte Carlo simulation. α is the significance level, α=0.05 .

$$UCL=\hat{d}F_{\alpha }\left(p,\hat{q}\right)$$ (5)

At this stage, we have derived robust Hotelling T^2^ statistics and established control limits based on the observations. When the statistic surpasses the control limit, an alarm signal is triggered. Conversely, if all the statistics fall within the control limit, the observations are considered to be in a normal state. Based on this, we can estimate the reliable OCRs and non-OCRs in the training set to reduce noise.

Because of the unique biological characteristics of biological data, OCRs cannot be assumed to occur once the robust Hotelling T^2^ control chart generates an alarm signal. Therefore, in order to effectively detect OCRs to obtain reliable training sets, we proposed three run rules for the detection of OCRs combined with Western Electric Rules (Cheng and Chou, 2008), which represent three possible cases of OCRs.

**FIGURE 3:**
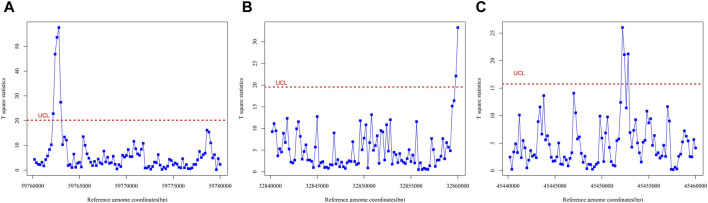
Hotelling T^2^ control charts on different reference genome fragments, representing each of the three cases in which open chromatin regions appear. Red lines are control limits. **(A)** Hotelling T^2^ control chart on reference genome fragment from 59760000 to 59780000, representing the occurrence of the OCR in run rule 1. **(B)** Hotelling T^2^ control chart on reference genome fragment from 32840000 to 32860000, representing the occurrence of the OCR in run rule 2. **(C)** Hotelling T^2^ control chart on reference genome fragment from 45440000 to 45460000, representing the occurrence of the OCR in run rule 3.

(1) Three or more consecutive observation points exceeded the control limit. After analyzing the length of the OCRs via ATAC-seq and DNase-seq, it was discovered that approximately 50% of the regions exceeded 300 bp in size, while over 25% exceeded 600 bp (Wang et al., 2021). For this study, a window size of 200 bp was selected, with each observation point representing that size. Specifically, two consecutive observations (400 bp in total) above the control limit were deemed to indicate an OCR. However, to account for noise interference in the original data, the error tolerance interval was set to one nucleosome size (approximately 167 bp). Consequently, OCR was defined as an area where three or more consecutive observations surpassed the control limits such as Figure 3A, based on the above considerations.
(2) The first or last point is outside the control limit. During data preprocessing, we truncated the reference genome into multiple segments. To detect OCRs that span across two segments, we established the criterion that if the first or last observation point in any monitored group exceeded the control limit, we would consider the region between the two observation points before and after that point (a total of 600 bp) as OCRs (Figure 3B).
(3) The number of points between two consecutive observations exceeding the control limit is less than three. The paper (Khoo, 2003) proposes two criteria for identifying out-of-control states: 1) If two out of the three most recent samples fall above the UCL or below the LCL on the control chart, the process is considered to be in an OC state. 2) If three out of the four most recent samples fall above the UCL or below the LCL on the control chart, the process is deemed to be in an OC state. Based on these criteria, we extended the analysis to include intervals with less than three points between adjacent points exceeding the control limits as indicative of an out-of-control state (Figure 3C).

#### 2.2.2 Design sensitized T^2^ control chart

By adhering to the aforementioned run rules, the robust Hotelling T^2^ control chart can effectively identify chromatin open regions and closed regions across the entire genome, thereby achieving the objective of filtering out noise labels from the training datasets. However, it is important to note that the robust Hotelling T^2^ control chart can only identify two states, namely OCRs and non-OCRs, and is unable to distinguish pOCRs. In order to accurately classify samples that contain a mixture of partially open states, we propose to use a sensitized T^2^ control chart specifically tailored to this scenario.

The monitoring statistic of the sensitized T^2^ control chart combines the probabilities from a multivariate control chart and a noise-tolerance classification model respectively and can be calculated as Formula 6:

$${ST}_{i}^{2}=\eta^{\delta \left(X_{i}\right)}\cdot p_{T^{2}}\left(X_{i}\right)+\left(1-\eta^{\delta \left(X_{i}\right)}\right)\cdot p_{C}\left(X_{i}\right)$$ (6)

where $X_{i}$ is the i th incoming query observation, and $p_{T^{2}}\left(X_{i}\right)$ and $p_{C}\left(X_{i}\right)$ are the probabilities that the observation $X_{i}$ belongs to the OCR class estimated from the MEWMA chart and OCRFinder model, respectively. Furthermore, η is the parameter that controls the amount of weight that we impose on the MEWMA chart. In this paper, the value of η is 0.3.

The first part of the monitoring statistic (i.e., $p_{T^{2}}\left(X_{i}\right)$ ) can be calculated using the following sigmoid function that returns values between zero and one, shown in Formula 7.

$$p_{T^{2}}\left(X_{i}\right)=\frac{1}{1+e^{-\left(f_{D}\left(X_{i}\right)\right)}}$$ (7)

where $f_{D}\left(x_{i}\right)$ can be computed as Formula 8:

$$f_{D}\left(X_{i}\right)=T^{2}-BD_{T^{2}}\left(\alpha ,B\right)$$ (8)

Here, $T^{2}$ is the typical monitoring statistic of MEWMA control chart, shown in Formula 9:

$$T^{2}=Z_{i}^{\prime }S_{Z}^{-1}Z_{i}$$ (9)

where $Z_{i}=\lambda X_{i}+\left(1-\lambda \right)Z_{i-1}$ and $S_{Z_{i}}=\frac{\lambda }{2-\lambda }S$ . In addition, 0 < λ≤1 and $Z_{0}=0$ . $BD_{T^{2}}\left(\alpha ,B\right)$ are the $100\left(1\;-\alpha \right)$ th percentile values from the non-OCRs $T^{2}$ statistics based on bootstrapping in which α is the user-defined Type I error rate and B is the number of bootstrap samples.

The second part of the monitoring statistic (i.e., $p_{C}\left(X_{i}\right)$ ) represents the probability of an observation being associated with one of the categories, and can be obtained using the following sigmoid function, shown in Formula 10:

$$p_{C}\left(X_{i}\right)=\frac{1}{1+e^{-\theta }}$$ (10)

where θ is the output of linear neural units.

Now we return to Eq. 6. Here, $\delta \left(X_{i}\right)$ indicates the confidence of the predicted value from the classification model, which can be determined using Equation 11:

$$\delta \left(X_{i}\right)=\left|0.5-p_{C}\left(X_{i}\right)\right|\cdot 2$$ (11)

Because the distribution of the sensitized T^2^ monitoring statistic is unknown, the control limit (CL) is determined from a percentile value estimated by bootstrapping. To enhance the effectiveness of identifying pOCRs, we have fine-tuned the pre-determined control limits by multiplying them with the factor of mixture ratio. This approach allows for a more accurate classification of the sample categories. By accommodating the mixture ratio, we can fully utilize the characteristics of partially open states and thus improve the classification performance of the training set.

### 2.3 Construct deep learning model based on confident learning framework

Although sensitized T^2^ control chart can classify OCRs, non-OCRs, and pOCRs to a certain extent in the training set, there may still be a few noisy labels in the classification results. In order to obtain more accurate classification results, we have introduced a machine learning model based on the confident learning framework (Curtis et al., 2021) after the sensitized T^2^ control chart. The confident learning framework can be divided into the following three steps: 1) estimating the joint distribution of noisy labels and true labels; 2) identifying erroneous samples and filtering them out; 3) adjusting the weights of the sample classes and retraining the model. In order to address the issue of noisy label overfitting, we have implemented a co-teaching approach (Han et al., 2018). Compared to training with noisy labels using a single model, this collaborative training method effectively reduces the overfitting impact of incorrect labels. This involves training two identical models and utilizing the clean data to guide each other during the back-propagation process. Specifically, in each iteration (e.g., the kth iteration), model A utilizes a clean dataset $D_{A}$ during the forward propagation, and then model B employs $D_{A}$ for backpropagation to update itself. The same process is reciprocated for model A’s update. By employing this co-teaching strategy, we indirectly mitigate the problem of overfitting that arises when a single model employs identical parameters for both prediction and update.

In this section, we convert the cfDNA-seq data into image-based inputs. Our model architecture comprises several key components, including a one-dimensional convolutional layer, a max-pooling layer, a bidirectional LSTM layer, another one-dimensional convolutional layer, another max-pooling layer, and finally a fully connected layer. In this paper, the deep learning model is called ConvLSTM. The focus of this study is on how to design a noisy label learning algorithm based on deep learning for OCRs estimation rather than feature extraction, the selection of deep learning models is not considered in this paper.

The OCRClassifier algorithm is designed to accurately classify data that contains a mixture of the third category labels, while only two labels are marked. The training dataset, obtained after the application of the robust Hotelling T^2^ control chart filtering, exclusively consists of reliable OCRs and non-OCRs data. This dataset is used to train a sensitized T^2^ control chart that is capable of recognizing three categories: chromatin open regions, partially open regions, and closed regions. For dataset including pOCRs, the sensitized T^2^ control chart can reassign labels and then the co-teaching model will produce the final accurate classification results.

## 3 Results

The cfDNA sequencing data of healthy individuals (IH01, IH02, BH01) used in this study were provided by Snyder (Snyder et al., 2016). The sequenced reads were aligned to the GRCh37 human reference genome. The sequencing coverage was 96-105x, with approximately 15–16 million sequencing fragments (Snyder et al., 2016). The OCRs of the hematopoietic lineage used in this study were obtained from the ENCODE database, specifically from the results of ATAC-seq and DNase-seq experiments. The housekeeping genes file, ATAC-seq experimental results, and DNase-seq experimental results were all obtained from Wang’s study (Wang et al., 2021).

In order to evaluate the performance of our algorithm, we adopt a similar approach as OCRFinder, relying on known gene expression levels or chromatin accessibility levels. For the initial training and test sets, we adopt the same selection approach as OCRFinder, chromosomes 2-7 are used for training, and chromosome 1 is used for testing. The OCR samples in initial training set are from housekeeping genes and non-OCRs samples are obtained by non-genetic regions. Specifically, the training set is filtered by the robust Hotelling T^2^ control charts based on MRCD estimation, and we get 800 OCR samples through this process. The principle of filtering is that if the OCR sample in the training set overlaps with the open area obtained by the robust Hotelling T^2^ control chart, the OCR sample is retained and others will be filtered out. The same filtering rules are applied to the non-OCR samples. Among these 800 OCR samples, we selected 400 to serve as OC information for training the sensitized T^2^ control chart. Additionally, we chose 1,000 samples from the filtered non-OCR samples to provide IC information for training the sensitized T^2^ control chart. Due to the unavailability of clearly labeled data for pOCRs, we simulate the required data $D_{POCR}$ by utilizing filtered chromatin open regions $D_{OCR}$ and closed regions $D_{CCR}$ , the Formula 12 is as follows:

$$D_{POCR}=D_{OCR}\cdot \tau +D_{CCR}\cdot \left(1-\tau \right)$$ (12)

where τ is the composite rate, unless otherwise stated, the value of τ is 0.7 in this paper. Since the simulated sequencing data is extracted from the real cfDNA-seq data, the research conducted on the simulated data could be appropriate for the real scenario. Indeed, it should be noted that the labels for partially open region data were randomly assigned either an open region label or a closed region label during the OCRClassifier training process.

For test set, we also selected highly expressed housekeeping genes as OCR samples (class 1) and non-genetic regions without gene expression as non-OCR samples (class 0) and simulate the pOCR samples (class 2). To exclude gene-specific chance, the results of ATAC-seq and DNase-seq experiments in hematopoietic lineage cells are combined. We conducted comparison experiments on three test sets: HK_TSS, Hematopoietic_Lineage_ATAC, Hematopoietic_lineage_DNase.

We used the accuracy (ACC), recall (REC), precision (PRE), F1 score (F1), area under the receiver operating characteristic curve (AUC) and area under the precision-recall curve (AUPR) as the criteria for the performance evaluation.

$$ACC=\frac{TP+TN}{P+N}=\frac{TP+TN}{TP+FN+TN+FP}$$

$$REC=\frac{TP}{P}=\frac{TP}{TP+FN}$$

$$PRE=\frac{TP}{TP+FP}$$

$$F1-\text{score}=2\times \frac{PRE\times REC}{PRE+REC}$$

During model training, we used the ConvLSTM network and selectd Adam optimizer (Kingma and Ba, 2014). The learning rate was set to 1e-4, the batch size was 128, and the training epoch was 150, with an additional warm-up training epoch of 10. It is important to note that the reported results in this paper are the average of five independent experiments, each initialized with a different random seed.

### 3.1 Effectiveness analysis of the robust Hotelling T^2^ control chart

Due to the dynamic nature of open chromatin regions in tissue-specific contexts, it is challenging to obtain a large number of reliable labeled samples for model training. OCRFinder is a noise-tolerance approach and has performed impressive results in various evaluations. However, it is observed that the performance of current noisy label learning methods tends to diminish as the proportion of noisy labels in the training set increases. Thus, the performance of the OCRFinder is still attenuated by the proportion of noisy labels in the training sets. In this study, we utilize the robust Hotelling T^2^ control chart to further filter out the noisy labels in the initial training dataset and then enhance the performance of the noise-tolerance model.

**FIGURE 4:**
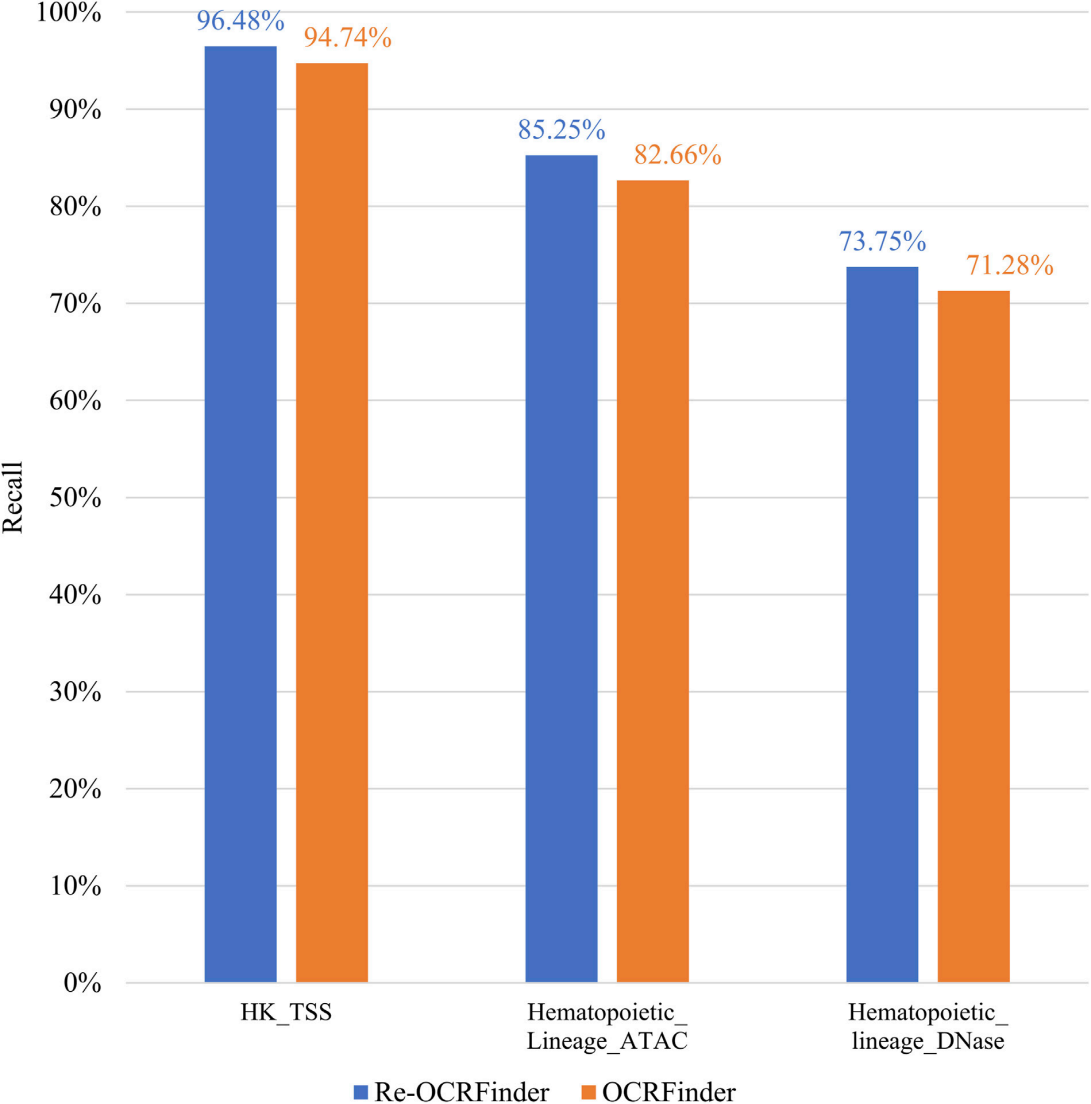
The comparison on sensitivity between retrained OCRFinder and OCRFinder on different testing sets.

As mentioned earlier, the original training dataset used for OCRFinder was filtered through robust Hotelling T^2^ control chart, resulting in 800 positive samples. To ensure a balanced dataset, an equal number of negative samples are also retained, resulting in a training set comprising a total of 1,600 samples. After retraining OCRFinder with the filtered training dataset, as shown in Figure 4, there has been an improvement in the model’s performance. Table 1 shows that the retrain model outperforms OCRFinder in both AUC and AUPR on each test set. From this perspective, it is evident that the robust Hotelling T^2^ control chart is capable of performing OCR recognition on the training dataset, thereby reducing noise percentage in the initial training set. This dual effect allows for an improved performance of the OCRFinder model in OCR recognition while also providing assurance for the training of a three-class sensitized T^2^ control chart recognition.

**TABLE 1:** The AUC and AUPR values of retrained OCRFinder and OCRFinder on different testing sets.

	Re-OCRFinder (%)		OCRFinder (%)	
	AUC	AUPR	AUC	AUPR
HK_TSS	**98.46**	**98.7**	97.57	97.81
Hematopoietic_Lineage_ATAC	**96.23**	**96.98**	94.98	95.43
Hematopoietic_Lineage_DNase	**91.98**	**93.69**	89.42	91.29

Bold values represent the results from the proposed method.

### 3.2 Effectiveness analysis of the detection capability of sensitized T^2^ control chart

**FIGURE 5:**
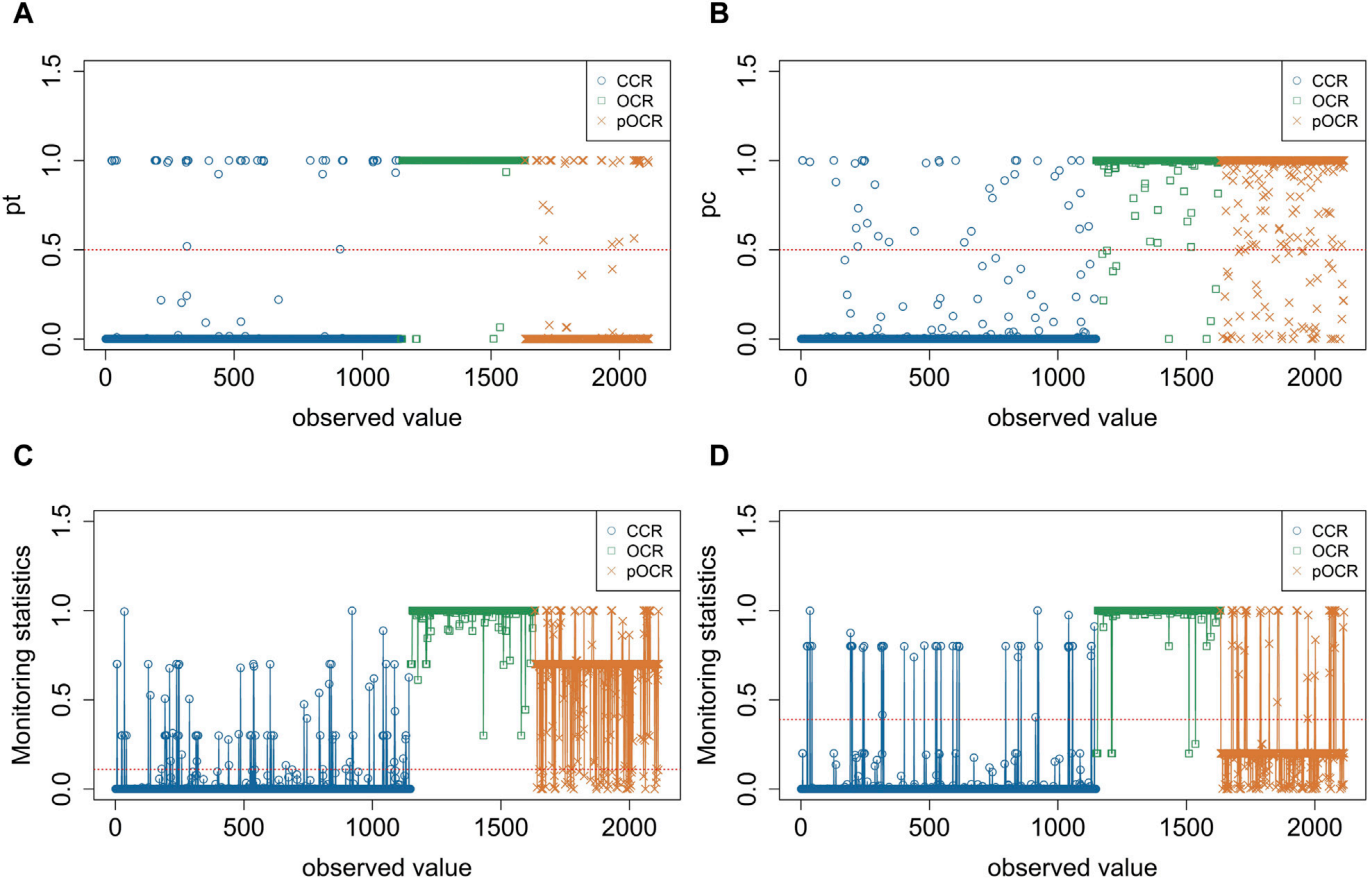
Performance of sensitized T^2^control chart. **(A)** MEWMA estimates the probability distribution of samples belonging to the open chromatin state. **(B)** OCRFinder estimates the probability distribution of samples belonging to the open chromatin state. **(C)** Statistical monitoring of sensitized T^2^ control chart ( η=0.3 ). **(D)** Statistical monitoring of sensitized T^2^ control chart ( η=0.8 ).

To verify the detection capability analysis of sensitized T^2^ control charts on partially open chromatin regions, we compared the classification results of EWMA, OCRFinder, and the designed sensitized T^2^ control chart. Traditional multivariate control charts can only identify two states: out-of-control and in-control. When the statistic exceeds the control limit, it is considered out-of-control, while it is considered in-control when the statistic is below the control limit. Similarly, the MEWMA multivariate control chart can only distinguish between OCRs (out-of-control) and non-OCRs (in-control), but it cannot differentiate pOCRs. $p_{T^{2}}$ represents the probability of the sample belonging to the OCR estimated by the MEWMA control chart. When $p_{T^{2}}\leq 0.5$ , it indicates that the sample is estimated to belong to the non-OCR; when $p_{T^{2}}>0.5$ , it indicates that the sample is estimated to belong to the OCR. Figure 5A shows the probability distribution of samples estimated to belong to the open chromatin state by the MEWMA control chart. For the OCRFinder binary classification model, $p_{C}$ represents the probability of the sample belonging to the open chromatin state estimated by the OCRFinder model. When $p_{C}\leq 0.5$ , it indicates that the sample is estimated to belong to the non-OCR; when $p_{C}>0.5$ , it indicates that the sample is estimated to belong to the OCR. Figure 5B shows the probability distribution of samples estimated to belong to the open chromatin state by the OCRFinder model. Therefore, relying solely on the MEWMA control chart or OCRFinder classification model cannot distinguish the pOCRs. The sensitized T^2^ control chart proposed in this study combines the MEWMA control chart and OCRFinder classification model, and its performance is shown in Figure 5C. It can be observed that the sensitized T^2^ control chart can roughly detect pOCRs. As shown in Figure 5C, samples are classified as CCRs when the value ${ST}^{2}\epsilon \left[0,CL\right]$ , categorized as pOCRs when ${ST}^{2}\left.\epsilon (CL,1-\eta \right]$ and fall into the OCRs category when ${ST}^{2}\left.\epsilon (1-\eta ,1\right]$ .

**FIGURE 6:**
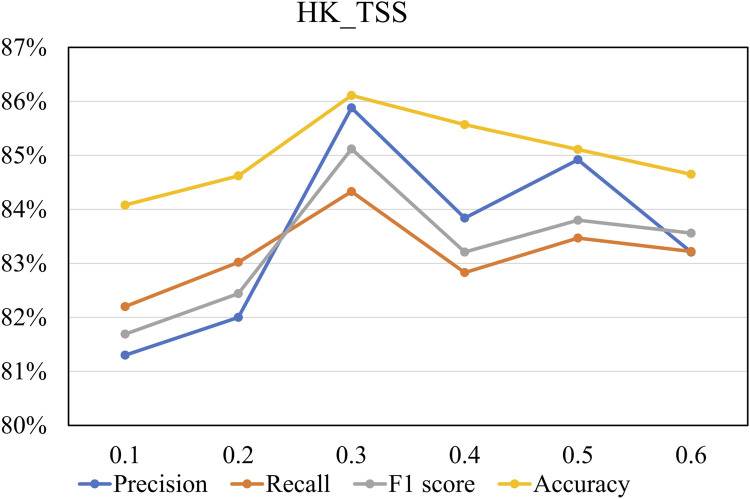
The performance on the HK_TSS dataset under different η values.

To determine the optimal value of η , the size of η varied from 0.1, 0.2, and 0.3 to 0.9 in the experiment. It should be noted that the control limits of the sensitized T^2^ control chart are indicators for distinguishing between in-control and out-of-control states. The non-OCR belongs to the in-control state, and its statistic should be below the control limit. The OCR and pOCR both belong to the out-of-control state, and their statistics should be above the control limit. However, when η exceeds 0.7, the statistics of the pOCRs are also below the control limit. Figure 5D shows the monitoring statistics for η=0.8 , where the red dashed line represents the control limit. It can be seen that only a very small portion of the pOCRs has statistics exceeding the control limit, while the majority are below the control limit, indicating that η ≥ 0.7 leads to inaccurate conclusions. Therefore, we only consider η≤0.6 in the following experiment. Figure 6 shows the performance of the OCRClassifier model on various evaluation metrics at different η values, using the HK_TSS dataset. From Figure 6, it can be observed that as η increases from 0.1 to 0.3, the model’s precision, recall, F1 score, and accuracy values all show an increasing trend. However, as η increases from 0.3 to 0.6, the model shows a general decline in performance across all metrics. Therefore, when η is set to 0.3, the model achieves optimal performance. Unless otherwise specified, the value of η is set to 0.3 in this paper.

### 3.3 Comprehensive performance analysis of OCRClassifier under different data sets

**FIGURE 7:**
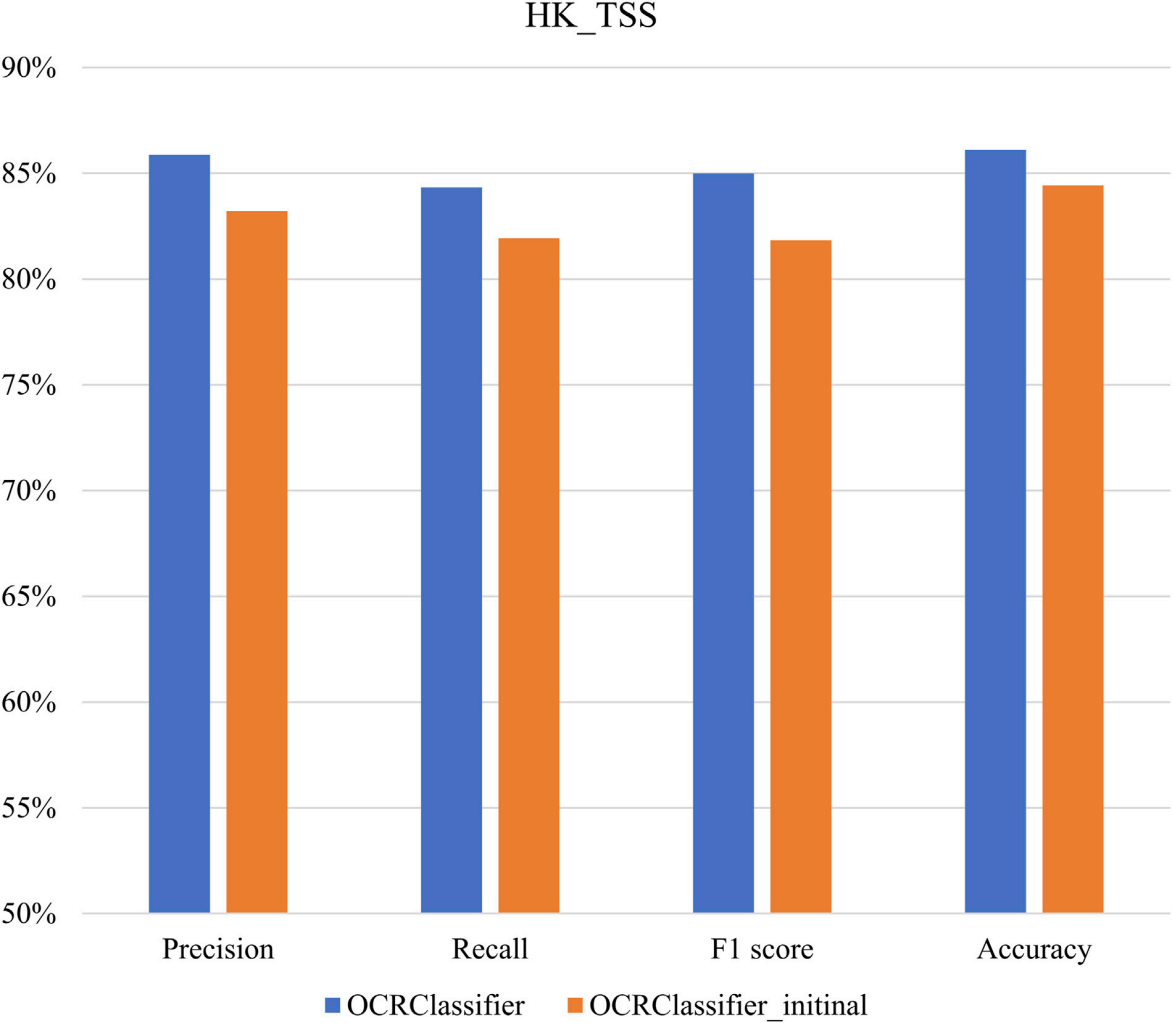
The performance of OCRClassifier on the HK_TSS dataset.

The primary objective of our study is to thoroughly evaluate the performance of the OCRClassifier algorithm using diverse reference datasets. In Figure 7, we present an extensive analysis of the OCRClassifier’s performance specifically on the HK_TSS dataset. The results reveal that the OCRClassifier has exceptional performance in identifying three essential categories: open chromatin regions, partially open regions, and closed chromatin regions. In four evaluation criterions, the OCRClassifier achieves the level above 84%. Moreover, we also provide the results from the OCRClassifier without the confident learning (OCRClassifier_initinal). Notably, the OCRClassifier outperforms OCRClassifier_initinal, exhibiting improvements of up to 3 percentage points across all evaluation metrics, demonstrating that incorporating confident learning significantly enhances the overall performance of the algorithm.

Table 2 presents a comprehensive overview of the performance of the OCRClassifier algorithm across different datasets. From the table, it is evident that the OCRClassifier consistently outperforms the OCRClassifier_initial in various metrics. Additionally, the HK_TSS dataset exhibits the highest model performance, followed by the Hematopoietic_Lineage_ATAC dataset, while the Hematopoietic_Lineage_DNase dataset lags behind. These results are consistent with established biological findings.

**TABLE 2:** The performance of the OCRClassifier algorithm across different datasets.

	OCRClassifier (%)				OCRClassifier_initial (%)			
	Precision	Recall	F1 score	Accuracy	Precision	Recall	F1 score	Accuracy
Hematopoietic_Lineage_ATAC	**78.54**	**74.2**	**74**	**74.2**	74.12	71.92	70.76	71.92
Hematopoietic_Lineage_DNase	**74.6**	**67.99**	**67.76**	**67.99**	70.41	66.18	65.04	66.18

Bold values represent the results from the proposed method.

### 3.4 Influence of mixture ratios on the performance of OCRClassifier

**FIGURE 8:**
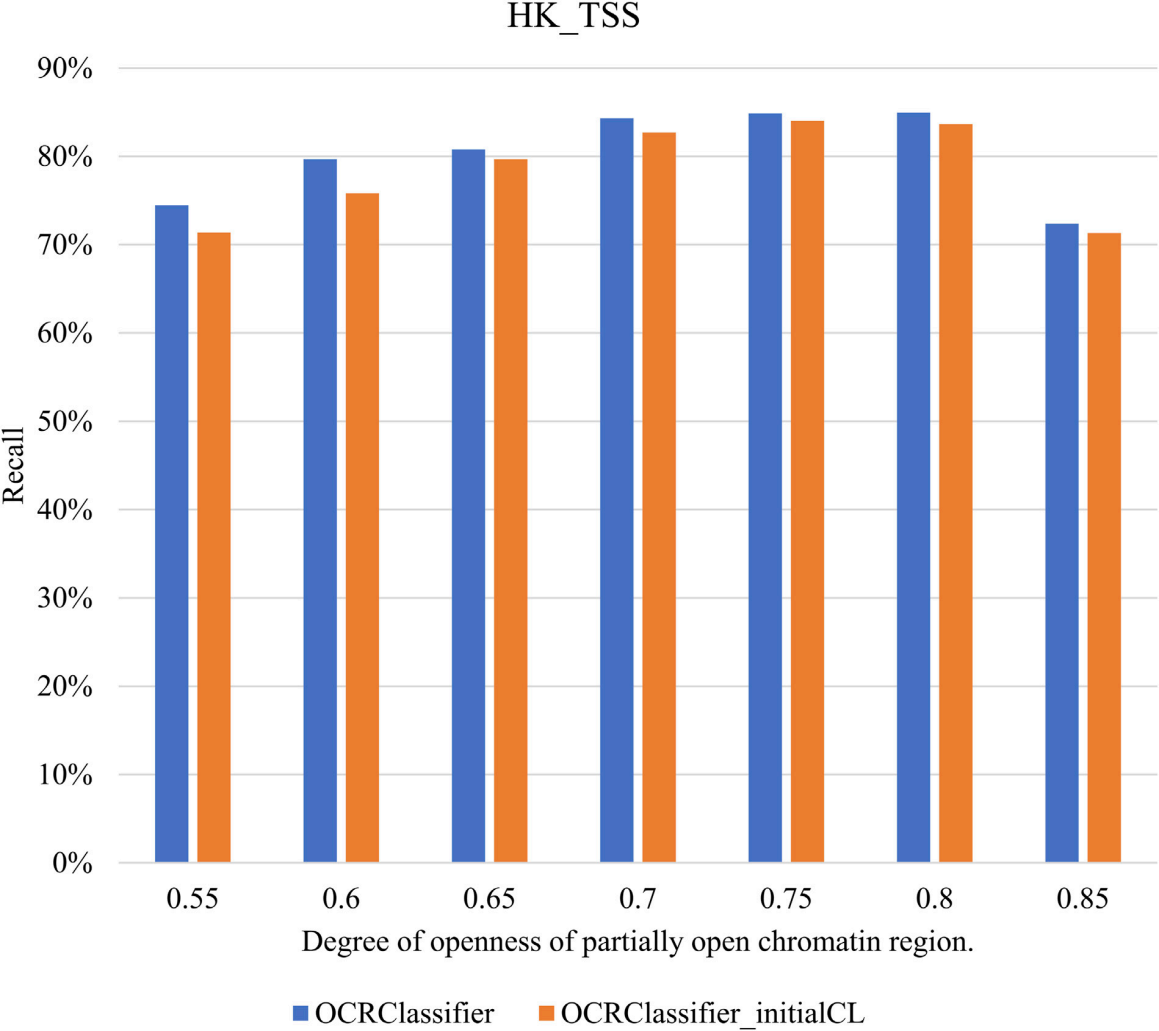
The classification performance under different mixture ratios on the HK_TSS dataset.

By varying the magnitude of the mixture ratio, the degree of openness of pOCRs can be altered. To investigate the impact of pOCRs openness on OCRClassifier model classification performance, we increased the values of the mixture ratio from 0.55 to 0.85. Then the relationship between pOCRs openness and model classification performance can be obtained. In Figure 8, the degree of openness of pOCRs is indicated by the mixture ratio. It could be observed that the variation of pOCR openness has no significant impact on OCRClassifier’s performance, suggesting the robustness of OCRClassifier. It should be noted that the model’s classification performance slightly decreased from 0.8 to 0.85. This could be the reason that the degree of openness of pOCRs at 0.85 becomes quite similar to that of OCRs, making it challenging for the sensitized T^2^ control chart to differentiate between these two states during the training set partitioning process.

Furthermore, this study made fine adjustments to the control limits of the sensitized T^2^ control chart. As depicted in Table 3, OCRClassifier‘s performance improved after these adjustments. Because pOCRs are positioned in an intermediate state, the sensitized T^2^ control chart becomes more effective in distinguishing pOCRs by refining the control limits, thereby enhancing the overall performance of the model.

**TABLE 3:** The sensitivity of OCRClassifier before and after the adjustment of control limits in sensitized T^2^ control chart on different datasets.

		0.55	0.6	0.65	0.7	0.75	0.8	0.85
HK_TSS	OCRClassifier (%)	**74.45**	**79.67**	**80.8**	**84.33**	**84.87**	**84.96**	**72.36**
	OCRClassifier_initialCL (%)	71.36	75.83	79.67	82.7	84.01	83.65	71.31
Hematopoietic_Lineage_ATAC	OCRClassifier (%)	**64.44**	**68.99**	**71.03**	**74.2**	**75.51**	**75.44**	**61.87**
	OCRClassifier_initialCL (%)	62.47	66.01	68.14	73.35	74.37	73.45	66.83
Hematopoietic_Lineage_DNase	OCRClassifier (%)	**60.28**	**63.74**	**65.63**	**67.99**	**68.58**	**68.48**	**62.09**
	OCRClassifier_initialCL (%)	58.54	61.47	63.3	67.13	67.83	66.5	61.93

Bold values represent the results from the proposed method.

### 3.5 Analysis of classification ability compared with OCRFinder

The OCRFinder model is a binary classification model that can only identify two categories: open and closed regions of chromatin. However, the proposed model OCRClassifier in this study is a three-classification model that not only identifies open and closed chromatin regions but also recognizes partially open regions. Nevertheless, both models share similarities in terms of their training datasets, which consist of samples labeled as either 0 or 1. The difference lies in the fact that the OCRClassifier’s training dataset includes a mixture of samples labeled as 2 in practice. To evaluate the classification performance of the OCRClassifier, we conducted two comparative experiments.

Firstly, we compared its binary classification ability, specifically, the capability to identify OCRs and non-OCRs, with that of the OCRFinder model. The performance results of the OCRClassifier and OCRFinder for different datasets are presented in the Table 4. From the table, the OCRClassifier exhibits a slightly lower classification ability compared to the OCRFinder when the test dataset only includes OCRs and non-OCRs. This finding aligns with practical expectations because the OCRClassifier is designed for a three-classification. When restricted to binary classification, the OCRClassifier model may misclassify certain samples as a third category, leading to a weaker performance than the OCRFinder.

**TABLE 4:** The comprehensive performance of the OCRClassifier and OCRFinder models across different training sets.

	OCRClassifier (%)				OCRFinder (%)			
	Precision	Recall	F1 score	Accuracy	Precision	Recall	F1 score	Accuracy
HK_TSS	**95.26**	90.36	93.21	91.15	94.32	**95.14**	**94.54**	**94.85**
Hematopoietic_Lineage_ATAC	91.15	81.23	85.25	81.69	**94.51**	**87.35**	**88. 65**	**88.92**
Hematopoietic_Lineage_DNase	**86.1**	77.2	79.5	76.81	85.01	**83.14**	**83**	**83.17**

Bold values represent better performance of the two models under the same metric.

**FIGURE 9:**
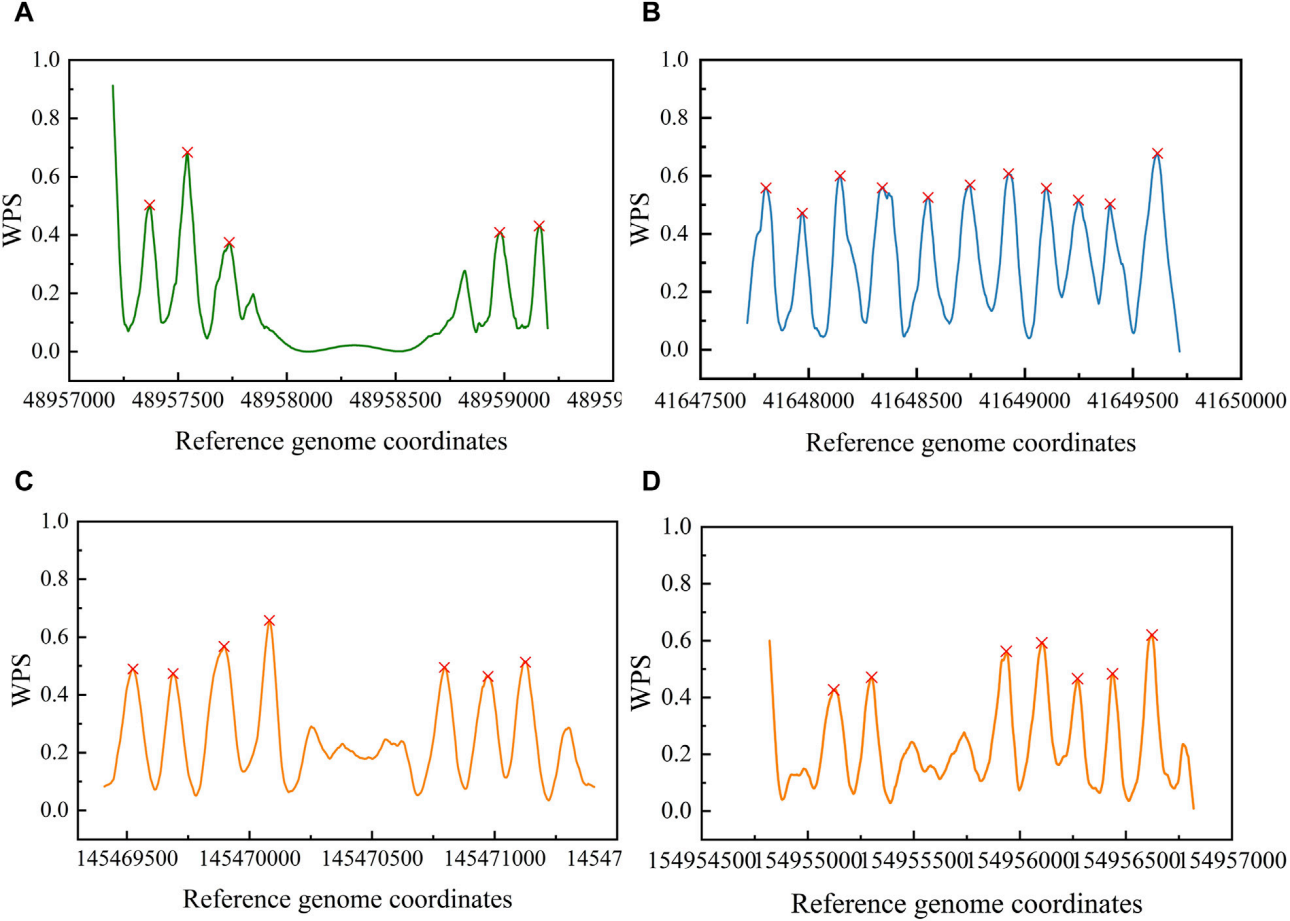
The classification results of different samples by OCRFinder and OCRClassifier models. **(A)** The OCR sample that was classified as OCR by both models. **(B)** The CCR sample that was classified as CCR by both models. **(C)** The pOCR sample that has been misclassified as OCR by OCRFinder. **(D)** The pOCR sample that has been misclassified as CCR by OCRFinder.

Then, we validated the OCRClassifier’s ability in recognizing the pOCRs. We applied OCRFinder to classify a test dataset that contained pOCRs. The results of the classification generated only labels 0 and 1. Simultaneously, we employed OCRClassifier to classify the same test dataset, resulting in labels 0, 1, and 2. Analyzing the classification results of OCRFinder, we removed the intersection of labels 0 and 1 from the two sets (samples like Figures 9A, B). After this removal, we found that some samples (like Figures 9C, D) were classified as label 2 by OCRClassifier, while OCRFinder misclassified them as label 0 or label 1, as depicted in the waveform Figure 9. Hence, it is evident that OCRClassifier exhibits the capability to recognize the third category pOCRs.

## 4 Discussion and conclusion

OCRs play a significant regulatory role in human biological activities and growth. The multi-class evaluation of the open state of chromatin regions can contribute to further cancer analysis research. Currently, methods for identifying chromatin open regions are limited to recognizing only two states: completely open and completely closed. For datasets that contain partially open chromatin regions, existing models may not be able to identify them. Additionally, training datasets based on biological experiments for OCRs and non-OCRs often include a high proportion of noisy labels, which substantially attenuates the model performance. By reducing the proportion of noisy labels in the training set, the identification ability of model could be further improved.

In this paper, we propose open chromatin region classifier (OCRClassifier), which can reduce the noisy labels in the initial training set and identify OCRs, non-OCRs, and pOCRs. The main task of OCRClassifier is to utilize robust Hotelling T^2^ control chart to filter the initial training set, which consists of only OCRs and non-OCRs, based on the different distributions of cfDNA-seq data features in different states. This filtering process aims to obtain more reliable samples for training, thereby enabling the development of a sensitized T^2^ control chart that can identify a third category of pOCRs. The experimental results demonstrate that OCRClassifier achieves a performance of over 84% in terms of accuracy, precision, recall, and F1-score, indicating its strong ability for three-class classification. Furthermore, the model exhibits high robustness in classification performance, which is unaffected by various openness levels of pOCRs. This framework allows for the improvement of OCRFinder in binary classification, by integrating the robust Hotelling T^2^ control chart with OCRFinder. Specifically, the OCRFinder is trained after filtering the training set by the robust Hotelling T^2^ control chart. The resulting model exhibits a 3-percentage point increase in sensitivity compared to the original OCRFinder. Additionally, the AUC and AUPR values of proposed method are also higher than original OCRFinder. The concept of OCRClassifier can be extrapolated to address label-unknown or label-ambiguous challenges in other bioinformatics domains as well, offering a promising approach to tackle the expensive annotation issues associated with biomedical samples.

The OCRClassifier is capable of identifying OCRs, non-OCRs, and pOCRs, where the data for pOCRs is simulated based on the other two categories. However, cfDNA is a composite of various cell types. Neutrophils, lymphocytes and liver are the primary contributors to cfDNA (Sun et al., 2015), resulting in the detection of a higher number of OCRs through cfDNA analysis, and our method can only detect these regions, independent of cell type. Therefore, the improvement of the OCRClassifier for predicting cell type-specific or tissue-specific OCRs and pOCRs is needed in future study.

## Data Availability

The original contributions presented in the study are included in the article/supplementary material, further inquiries can be directed to the corresponding author.
